# Adipose‐Derived Stem Cells Differentiate Into Insulin‐Producing Cells in 2D Culture With Photobiomodulation: A Comparative Analysis of Wavelength and Fluence Parameters

**DOI:** 10.1002/cbin.70182

**Published:** 2026-06-30

**Authors:** O. Daramola, H. Abrahamse, A. Crous

**Affiliations:** ^1^ Laser Research Centre, Faculty of Health Sciences University of Johannesburg Doornfontein South Africa

**Keywords:** adipose‐derived stem cells, diabetes, functional insulin‐producing β cells, photobiomodulation, two‐dimensional culture conditions

## Abstract

Diabetes mellitus is a chronic metabolic disorder characterized by the loss or dysfunction of insulin‐producing beta (β) cells. Adipose‐derived stem cells (ADSCs) represent a promising source for generating functional insulin‐producing β cells due to their accessibility and differentiation potential. Photobiomodulation (PBM), a non‐invasive light‐based therapy, has emerged as an innovative strategy to enhance stem cell differentiation efficiency. Evidence suggests that green (525 nm) and near‐infrared (825 nm) wavelengths, applied individually or in combination, can modulate cellular metabolism, ATP production, and differentiation‐related signaling pathways, thereby influencing ADSC commitment toward insulin‐producing β‐cell‐like phenotypes. This in vitro study evaluated the effects of PBM at 525 nm and 825 nm, delivered individually and in combination at energy fluences of 5 J/cm^2^ and 10 J/cm^2^, on the differentiation of ADSCs cultured in β‐cell induction medium into insulin‐producing β‐cell‐like cells under two‐dimensional (2D) culture conditions at 24 h, 5 days, and 10 days. Cellular responses were evaluated using adenosine triphosphate (ATP) luminescence assays, lactate dehydrogenase (LDH) activity assays, Giemsa staining, Live/Dead viability assays, and dithizone (DTZ) staining. ATP levels varied significantly among the experimental groups, reflecting changes in cellular metabolic activity associated with β‐cell induction and PBM exposure, and reduced LDH activity, suggesting decreased cytotoxicity. Giemsa staining revealed morphological changes consistent with β‐cell differentiation, while Live/Dead assays demonstrated the maintenance of cell viability across all experimental groups. Dithizone staining identified the presence of zinc‐rich insulin‐producing clusters. These findings highlight the importance of PBM wavelength and fluence optimization in regenerative stem cell applications.

**Abbreviations**
2D: two‐Dimensional
β‐cell: beta cell
ADSC(s): adipose‐derived stem cell(s)
AO: acridine orange
AO/EB: acridine orange/ethidium bromide
ATP: adenosine triphosphate
BSA: bovine serum albumin
CO_2_
: carbon dioxide
DMEM: Dulbecco's modified eagle medium
DMEM/F‐12: Dulbecco's modified eagle medium/Ham's F‐12 Medium
DMSO: dimethyl sulfoxide
DNA: deoxyribonucleic Acid
DTZ: dithizone
EB: ethidium bromide
ELISA: enzyme‐linked immunosorbent assay
FBS: fetal bovine serum
GLP‐1: glucagon‐like peptide‐1
iADSC(s): immortalized adipose‐derived stem cell(s)
ITS: insulin‐transferrin‐selenium
J/cm^2^
: Joules per Centimeter Square (Fluence)
LDH: lactate dehydrogenase
NA: numerical aperture
NIR: near‐infrared
nm: nanometer
PBM: photobiomodulation
PBS: phosphate‐buffered saline
RLUs: relative light units
SEM: standard error of the mean
USA: United States of America
VAC: volts alternating current

## 1 Introduction

Diabetes mellitus, a common metabolic condition, is defined by the progressive loss or dysfunction of insulin‐producing beta (β) cells in the pancreas (Diane et al. 2022). Although exogenous insulin administration and pancreatic islet transplantation are two current treatment approaches that can lessen the effects of the disease, they have important drawbacks (Rao et al. 2020). Insulin therapy cannot completely replicate physiological glucose regulation and is a lifelong treatment (Siwakoti et al. 2023), while long‐term graft failure, immunological rejection, and donor shortage are obstacles to islet transplantation (Jeyagaran et al. 2022). These drawbacks highlight how urgently alternative treatments that can restore endogenous insulin production are needed.

Stem cell‐based techniques are now a practical means of generating new β cells (Addissouky et al. 2024). Adipose‐derived stem cells (ADSCs), a type of mesenchymal stem cell found in adipose tissue, have attracted considerable interest because of their abundance, ease of isolation, and ability to differentiate into multiple cell lineages, including pancreatic β‐cell‐like cells (Miana and Prieto González 2018; Sowa et al. 2022). Consequently, ADSCs represent a potential source of insulin‐producing cells for replacing or repairing damaged β cells in individuals with diabetes (Jun and Park 2009). In addition to their differentiation potential, ADSCs possess immunomodulatory properties that may contribute to improved transplantation outcomes and regenerative responses (Papadopoulos et al. 2024). Their availability through minimally invasive harvesting procedures further enhances their therapeutic appeal for regenerative medicine applications (Papadopoulos et al. 2024). Research on the complex process of converting ADSCs into functional β cells has provided valuable insights into the potential of stem cell‐based treatments for diabetes (Zuo et al. 2025); however, important challenges remain, including optimizing differentiation efficiency, enhancing β‐cell maturation and functionality, achieving glucose‐responsive insulin secretion comparable to native pancreatic β‐cells, and validating long‐term safety and therapeutic efficacy in clinically relevant models (Yuan et al. 2024; Daramola et al. 2025).

Photobiomodulation (PBM) is a non‐invasive light‐based therapy that utilizes specific wavelengths to modulate cellular activity and has been reported to enhance cell viability, proliferation, and lineage‐specific differentiation in various stem cell populations, including adipose‐derived stem cells (Chang et al. 2020; Dompe et al. 2020). In ADSCs, PBM has been shown to influence cellular metabolism, proliferation, and differentiation toward specialized phenotypes, highlighting its potential as an adjunctive strategy for regenerative medicine applications (Dompe et al. 2020; Daramola et al. 2025). Photobiomodulation utilizes visible and near‐infrared wavelengths, typically ranging from 400 to 1100 nm, to influence cellular and biological processes, with treatment outcomes dependent on parameters such as wavelength, irradiance, exposure time, and fluence (Chung et al. 2012; Dompe et al. 2020). Previous studies have reported that PBM at fluences between 3 and 5 J/cm^2^ can enhance cellular proliferation, metabolic activity, and differentiation‐related responses in various cell types (Kingham et al. 2007; Wang et al. 2017a; Abrahamse and Crous 2024). The fluences of 5 J/cm^2^ and 10 J/cm^2^ were selected to investigate potential dose‐dependent effects of PBM, as previous studies have demonstrated that fluences within this range can modulate stem cell proliferation, metabolism, and differentiation‐related responses in accordance with the biphasic dose‐response characteristics of PBM (Hamblin 2018; Abrahamse and Crous 2024; Mulaudzi et al. 2026). Also, PBM is a novel strategy for boosting stem cell differentiation because of its ability to modulate mitochondrial activity and cellular energy metabolism (Hu et al. 2007; Hamblin 2018). In addition to enhancing cellular metabolism, PBM has emerged as a promising differentiation‐promoting strategy because it can regulate cellular signaling pathways associated with proliferation and differentiation (De Freitas and Hamblin 2016; Dompe et al. 2020). Furthermore, studies have shown that exposure to shorter visible wavelengths, such as blue (approximately 415–450 nm) and green (approximately 540 nm) light, may inhibit adipose‐derived stem cell proliferation while promoting osteogenic differentiation, highlighting the wavelength‐dependent nature of PBM effects (Wang et al. 2017b). In contrast, the proliferation of ADSCs has been reported to be enhanced by longer wavelengths, such as red (660 nm) and near‐infrared (810 nm) light (Wang et al. 2017b). Although cytochrome c oxidase exhibits absorption peaks within the red spectrum (approximately 630–670 nm) and near‐infrared region (approximately 780–940 nm) (Shen et al. 2024), the present study focused on 525 and 825 nm because previous studies have demonstrated that these wavelengths can modulate adipose‐derived stem cell differentiation and cellular activity in a wavelength‐dependent manner, either individually or in combination (Da Silva et al. 2023; Abrahamse and Crous 2024). This study therefore assessed the effects of PBM at a single green (G) wavelength of 525 nm, an NIR wavelength of 825 nm, and a combination of both wavelengths at fluencies of 5 J/cm2 and 10 J/cm2 under 2D culture conditions on the cellular morphology, proliferation, viability, cytotoxicity, and differentiation of ADSCs into functional insulin‐producing β cells.

## 2 Materials and Techniques

### 2.1 Model for Experimentation

**Figure 1:**
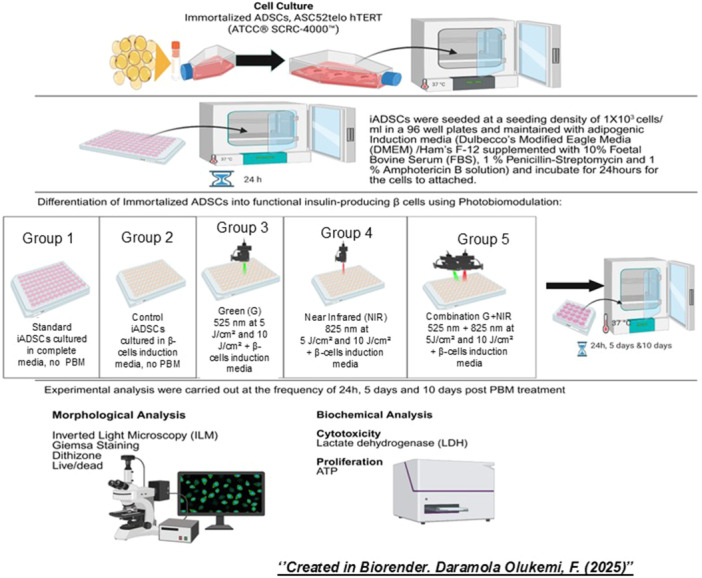
Schematic representation of the experimental design. iADSCs were revived and subcultured until the desired confluency was achieved before being cultured in β‐cell induction medium to promote differentiation toward a β‐cell‐like phenotype. Cells were exposed to PBM at wavelengths of 525 nm (green), 825 nm (near‐infrared), or a combination of both wavelengths at fluences of 5 J/cm^2^ and 10 J/cm^2^. Experimental groups included an induction control group cultured in β‐cell induction medium without PBM exposure and a baseline control group cultured in complete growth medium without β‐cell induction or PBM exposure. Samples were collected at 24 h, 5 days, and 10 days following PBM treatment. Cellular morphology was assessed using inverted light microscopy and Giemsa staining. Dithizone (DTZ) staining was used to evaluate the presence of zinc‐containing granules associated with β‐cell‐like differentiation, while Live/Dead staining was performed to assess cell viability. Cellular metabolic activity and cytotoxicity were evaluated using ATP luminescence and LDH activity assays, respectively.

Five different experimental groups were used to set up this experiment (Figure 1): Group 1 consisted of ADSCs cultured in complete growth medium in 96‐well plates under standard culture conditions, without exposure to PBM or β‐cell induction. This group served as a baseline control to enable comparison with induced cells and to verify the effects of the β‐cell induction protocol used in Group 2. The cells in Group 2 (induction control group) were cultured in 96‐well plates and maintained in β‐cell induction medium without exposure to PBM. Group 3 consisted of ADSCs cultured in β‐cell induction medium and exposed to PBM at a wavelength of 525 nm with fluences of 5 and 10 J/cm^2^. Group 4 consisted of ADSCs cultured in β‐cell induction medium and exposed to PBM at a wavelength of 825 nm with fluences of 5 and 10 J/cm^2^. Lastly, Group 5 consisted of ADSCs cultured in β‐cell induction medium and exposed to a combination of PBM wavelengths (525 nm and 825 nm) at fluences of 5 and 10 J/cm^2^.

### 2.2 Cell Culture

The hTERT immortalized ADSCs (SCRC‐4000, American Type Culture Collection, Manassas, VA, USA) were cultured in Corning cell culture flasks (CLS431080, Sigma‐Aldrich) ASC52telo in Dulbecco's Modified Eagle Media (DMEM) (D5796, Sigma‐Aldrich, Johannesburg, South Africa) supplemented with 10% fetal bovine serum (FBS) (S0615, Biochrom, Cape Town, South Africa) and 1% antibiotics: 0.5% Penicillin‐Streptomycin (Sigma‐Aldrich, P4333) and 0.5% Amphotericin B solution (A2942, Sigma‐Aldrich) at 37°C with 5% CO2 and 85% humidity (HeracellTM 150i CO2 Incubator, 51026280, Thermo Fisher Scientific, Johannesburg, South Africa). Upon reaching confluence, immortalized ADSCs (iADSCs) were seeded in 96 well culture plates (NunclonTM Delta Surface, Thermo Fisher Scientific, Roskilde, Denmark) at a density of 1 × 10^3^ cells. To allow cell attachment to the 96‐well culture plates, Groups 1–5 were maintained in 200 μL of complete growth medium and incubated for 24 h (Figure 1).

### 2.3 Beta Cells Induction

To initiate β‐cell induction, iADSCs were cultured in β‐cell induction medium consisting of DMEM/Ham's F‐12 (Thermo Fisher Scientific, Waltham, MA, USA) supplemented with 17.5 mM glucose (Inqaba Biotechnical Industries (PTY) Ltd., Hatfield, South Africa), 1% bovine serum albumin (BSA) (Roche Diagnostics, Indianapolis, IN, USA), 4 nM activin A (Biocom Africa (PTY) Ltd., Centurion, South Africa), 1 mM sodium butyrate (Inqaba Biotechnical Industries (PTY) Ltd., Hatfield, South Africa), 50 µM 2‐mercaptoethanol (Sigma‐Aldrich, St. Louis, MO, USA), and 1x insulin‐transferrin‐selenium (ITS) (Merck Life Science (PTY) Ltd., Aston Manor, South Africa) for 48 h (Chandra et al. 2009). After 48 h, the medium was replaced with serum‐free DMEM/Ham's F‐12 media (Thermo Fisher Scientific, Waltham, MA, USA) including 1% BSA (Roche Diagnostics, Indianapolis, IN, USA), 1x ITS (Merck Life Science (PTY) Ltd., Aston Manor, South Africa), and 0.3 mM taurine (Inqaba Biotechnical Industries (PTY) Ltd., Hatfield, South Africa), and the cells were incubated for an additional 48 h (Chandra et al. 2009). To initiate the final stage of β‐cell induction, the medium was replaced with serum‐free DMEM/Ham's F‐12 media (Thermo Fisher Scientific, Waltham, MA, USA) that contained 1.5% BSA (Roche Diagnostics, Indianapolis, IN, USA), 1x ITS (Merck Life Science (PTY) Ltd., Aston Manor, South Africa), 1 mM nicotinamide (Inqaba Biotechnical Industries (PTY) Ltd., Hatfield, South Africa), 3 mM taurine (Inqaba Biotechnical Industries (PTY) Ltd., Hatfield, South Africa), 100 nM human Glucagon‐Like Peptide (GLP)−1 (amide fragment 7‐36) (Biocom Africa (PTY) Ltd., Centurion, South Africa), and 1x non‐essential amino acids (Thermo Fisher Scientific, Waltham, MA, USA). Cells were maintained in this medium for 120 h, with medium changes performed every 48 h (Chandra et al. 2009).

## 3 Photobiomodulation

**Figure 2:**
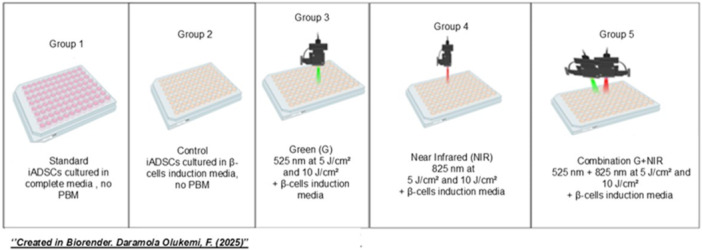
Shows the parameters for photobiomodulation and the experimental model.

Group 1 received 200 µL of complete growth medium, while Groups 2–5 received 200 µL of β‐cell induction medium. Cells were seeded at a density of 1 × 10^3^ cells per well in 96‐well culture plates (Nunclon™ Delta Surface, Thermo Fisher Scientific, Roskilde, Denmark) (Figure 2). A 525 nm diode laser (National Laser Centre, EN 60825‐1:2007) coupled to a 100–240 VAC, 47–63 Hz, 5 A laser source (OptoElectronics Tech. Co. Ltd., Changchun, China) and an 825 nm diode laser (SN 070900108, National Laser Centre, Pretoria, South Africa) coupled to a 1000 mA laser source (4210, Arroyo Instruments, CA, USA) were used to irradiate the attached iADSCs in Groups 3–5 (Figure 2). The laser output power (mW) was measured using a calibrated Field Mate Laser Power Meter (1098297, Coherent, Johannesburg, South Africa) coupled to a High‐Sensitivity Thermopile Sensor PM3 (1098336, Coherent). Irradiance (mW/cm^2^) was calculated by dividing the measured output power by the illuminated area (spot size), while irradiation time (s) was calculated from the desired fluence (J/cm^2^) and irradiance according to standard photobiomodulation dosimetry equations. The equations used for these calculations are shown below:

$$\mathrm{mW}/{\mathrm{cm}}^{2}=\frac{\mathrm{mW}}{\pi \times r^{2}}$$

$$W/{\mathrm{cm}}^{2}=\frac{\mathrm{mW}/{\mathrm{cm}}^{2}}{1000}$$

$$\mathrm{Time}\;\left(s\right)=\frac{J/{\mathrm{cm}}^{2}}{W/{\mathrm{cm}}^{2}}$$

Hence, mW/cm^2^ = power density (irradiance), W/cm^2^ = intensity, J/cm^2^ = fluence and s = exposure time.

The laser spot size was 9.62 cm^2^, corresponding to an illuminated beam diameter of approximately 3.50 cm, which fully covered the irradiated wells and cell monolayer. Photobiomodulation was delivered using a top‐illumination approach in the presence of culture medium. The reported irradiance and fluence values were calculated from the measured laser output power at the aperture and illuminated area. Light attenuation by the overlying culture medium was not measured directly and was therefore not incorporated into the dosimetry calculations. Cells were exposed to PBM once at the commencement of the experiment according to their assigned wavelength and fluence group. Due to the beam size, four wells of the 96‐well plate were irradiated simultaneously, while the remaining wells were shielded with sterile laminated paper to prevent unintended light exposure and maintain sterility. This procedure was repeated until all designated wells had received the prescribed irradiation dose. All irradiation parameters, including wavelength, output power, irradiance, spot size, fluence, and exposure time, are summarized in Table 1.

**Table 1:** Parameters of photobiomodulation.

Laser parameter	Green	Near‐infrared
Light source	Diode laser	Diode laser
Wavelength (nm)	525	825
Power output (mW)	490	127
Power density (mW/cm^2^)	53.99	13.99
Spot size (cm^2^)	9.62	9.62
Emission	Continuous wave	Continuous wave
Fluence (J/cm^2^)	5 and 10	5 and 10
Time of irradiation (s)	92 and 185	357 and 715

Hence, mW/cm^2^ = power density, W/cm^2^ = intensity, and s = exposure time.

## 4 Morphology

### 4.1 Inverted Light Microscopy

The cellular morphology of immortalized adipose‐derived stem cells (iADSCs) subjected to PBM was assessed using an inverted light microscope (Leica Mica Microhub, Leica Microsystems GmbH, Germany; distributed by Promolab Pty Ltd., South Africa). This advanced imaging system combines automated brightfield and fluorescence capabilities, allowing for high‐resolution visualization of live cells within a controlled environment. Brightfield imaging was carried out with a 10×/0.30 NA Plan Achromat objective lens, ensuring consistent exposure, light intensity, and focus across all samples to maintain uniformity. Image analysis was performed using Leica LAS X software (version X.X), with 250 µM scale bars added for standardized measurements.

### 4.2 Giemsa

Giemsa staining was used to assess the cellular morphology of ADSCs during β‐cell induction. This included evaluating cell size, shape, cellular arrangement, and cytoplasmic and nuclear characteristics, which provided morphological evidence consistent with cellular differentiation. However, Giemsa staining alone cannot definitively confirm differentiation into insulin‐producing β cells. The cells were first rinsed with 1× phosphate buffered saline (PBS), then fixed with methanol for 5 min, rinsed again with 1× PBS, stained for 4 min with May–Grünwald stain, after which the stain was removed, then stained for 6 min with Giemsa stains, before being rinsed three times with 1× PBS. A digital camera attached to the microscope (SC30, OLYMPUS) that uses the cellSens Imaging Software (OLYMPUS) was used to take pictures of the cellular morphological changes, which were viewed using inverted light microscopy (CKX41, OLYMPUS, Cape Town, South Africa).

### 4.3 Dithizone

Since β‐insulin‐producing cells have high zinc levels, they can be identified using the zinc‐chelating dye dithizone (DTZ) (43820‐10 G Merck Life Science (PTY) Ltd., Aston Manor, South Africa). The DTZ dye, which chelates zinc, was used to stain the cells. Dimethyl sulfoxide (DMSO) (Inqaba Biotechnical Industries (PTY) Ltd., Hatfield, South Africa) is used to dissolve 10 mg of DTZ in 1 mL; this creates the DTZ stock solution. Using 10 µL of the stock solution in 10 ml of culture medium, the cells were stained with DTZ, then incubated for 15 min at 37°C. After three PBS washes, the cells were examined under inverted light microscopy (CKX41, OLYMPUS, Cape Town, South Africa) and captured on camera using a digital camera attached to the microscope (SC30, OLYMPUS) that uses the cellSens Imaging Software (OLYMPUS).

### 4.4 Cell Viability: Live/Dead Assay

Acridine orange (AO) and ethidium bromide (EB) were used in a live/dead experiment to evaluate cell viability. Because it binds to nucleic acids, acridine orange can enter both living and dead cells, coloring them all green. However, by intercalating with DNA, ethidium bromide, which can only enter cells with damaged membranes, reddens dead cells. A density of 1 × 10^3^ cells was used to seed the cells in 96‐well culture plates. After the cells’ supernatant was extracted, any remaining media was cleaned out three times using 1× PBS. The AO & EB staining solution was then added to the cell suspension in 2 µL. At room temperature, the cells were shielded from light and the dye mixture was incubated for 5 min. The cells were rinsed three times with 1× PBS. Images were acquired using a Leica Mica Microhub, Leica Microsystems GmbH, Germany; distributed by Promolab Pty Ltd., South Africa, fluorescence microscope. Acridine orange created green fluorescence in living cells, whereas ethidium bromide staining caused red fluorescence in dead cells.

## 5 Biochemical Analysis

### 5.1 Proliferation: Adenosine Triphosphate (ATP) Luminescence Assay

CellTiter‐Glo 2.0 (G9241, Promega, Johannesburg, South Africa) adenosine triphosphate (ATP) luminescence assay was used to measure cell proliferation. Luminescent intensity was measured using relative light units (RLUs) and the Victor NIVO Multilabel Plate Counter (HH3522019094, PerkinElmer, Johannesburg, South Africa).

### 5.2 Cytotoxicity: Lactate Dehydrogenase

The CytoTox96 Non‐Radioactive Cytotoxicity Assay (G1780, Promega) was used to quantify lactate dehydrogenase (LDH) release as an indicator of cell membrane integrity and cytotoxicity. LDH is released into the culture medium following disruption of the plasma membrane. A lysis buffer‐treated positive control supplied with the assay kit was included to induce maximum LDH release and verify assay performance. Absorbance was measured at 490 nm using a Victor NIVO Multilabel Plate Counter, where the formation of a red formazan product is proportional to the amount of LDH released into the culture medium.

## 6 Statistical Analysis

The in vitro investigations were carried out four times (*n* = 4), and the experimental findings are shown as mean ± standard error of the mean (mean ± SEM). Graph Prism (version 9.3.1) software was used to statistically evaluate the in vitro collected data in order to determine the statistical significance. Tukey's post hoc test and two‐way analysis of variance (ANOVA) at a confidence interval of 95% were used to compare the standard, control, and treatment groups for statistical significance. Statistical significance, which was set at *p* < 0.05, was also established using ^#^ *p* < 0.05, ^##^ *p* < 0.01, ^###^ *p* < 0.001, and ^####^ *p* < 0.0001.

## 7 Results

### 7.1 Morphology (Inverted Light Microscopy, Giemsa, Dithizone and Live/Dead Assay)

#### 7.1.1 Inverted Light Microscopy

**Figure 3:**
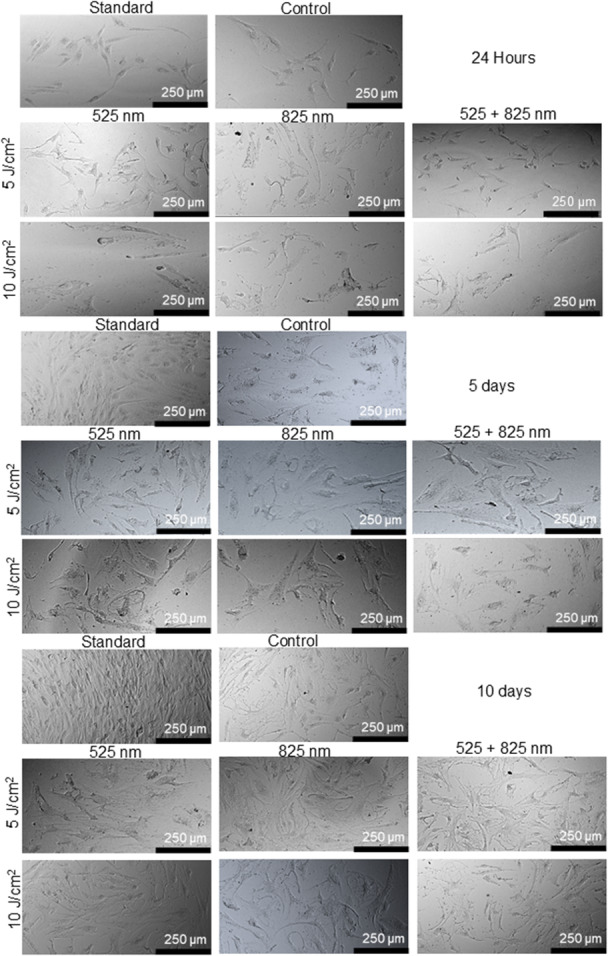
Representative phase‐contrast photomicrographs showing the morphology of iADSCs cultured under β‐cell induction conditions and exposed to PBM at 5 and 10 J/cm^2^. Images were acquired using a Leica MICA microscope at 10× objective at 24 h, 5 days, and 10 days following PBM treatment. Morphological changes associated with cellular differentiation were assessed over the experimental period. Scale bar = 250 µM.

Data were collected at 24 h, 5 days, and 10 days to evaluate the morphology of iADSCs differentiating into insulin‐producing β cells using a Leica MICA microscope with a 10× objective (Figure 3).

At 24 h, cells in the Group 1 (standard) and Group 2 (control group) retained their typical spindle‐shaped, fibroblast‐like morphology. Groups 3–5 (PBM‐treated groups) exhibited morphological changes characterized by increased cell spreading, extended cytoplasmic projections, and more clearly defined cell borders. Cells exposed to 10 J/cm^2^ generally exhibited lower cell density and reduced cytoplasmic extension compared with those exposed to 5 J/cm^2^.

By Day 5, morphological differences among the treatment groups became more apparent. Cells in Group 1 maintained a spindle‐shaped morphology with moderate growth, whereas those in Group 2 exhibited increased confluency and elongation. Groups 3–5 exposed to 5 J/cm^2^ exhibited increased cell density, elongated, spindle‐shaped morphology, and more extensive cellular networks. In contrast, cells treated with 10 J/cm^2^ exhibited reduced cell density and less pronounced elongation.

By Day 10, Group 1 remained predominantly spindle‐shaped and reached near confluency. The Group 2 exhibited polygonal cells and small cellular clusters. Cells exposed to PBM at 5 J/cm^2^, particularly Group 5, formed dense cellular clusters with polygonal morphology and minimal intercellular spacing. Groups 3–5 at 10 J/cm^2^ also formed clusters but exhibited lower density and less organized cellular arrangements than those observed at 5 J/cm^2^.

#### 7.1.2 Giemsa Stain

**Figure 4:**
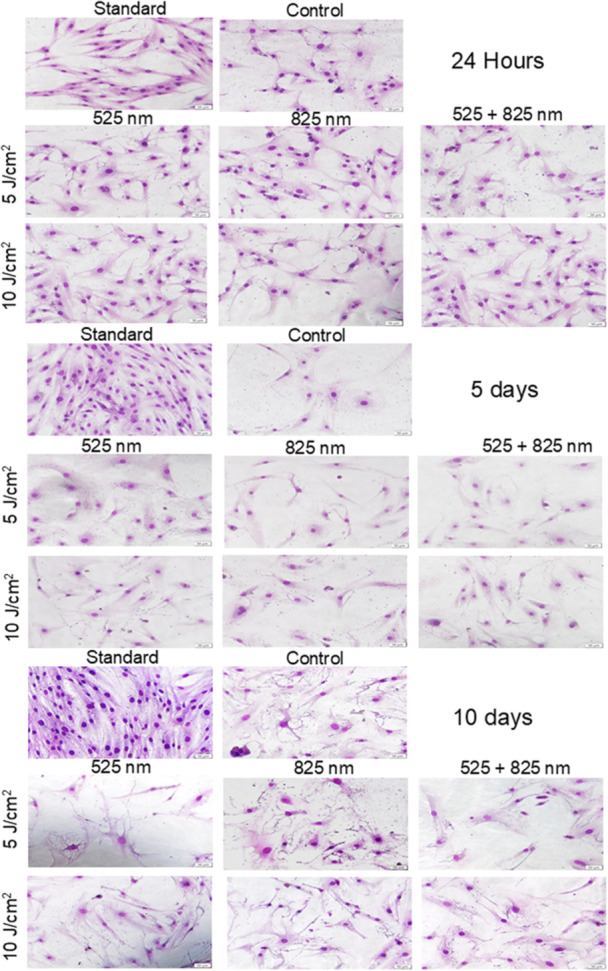
Representative photomicrographs of Giemsa‐stained iADSCs cultured under β‐cell induction conditions and exposed to PBM at fluences of 5 and 10 J/cm^2^. Images were acquired using an inverted light microscope at 20× objective at 24 h, 5 days, and 10 days following PBM treatment. Giemsa staining was used to assess cellular morphology, including cell shape, cellular arrangement, and cytoplasmic and nuclear characteristics during the differentiation process. Scale bar = 50 µm.

Giemsa staining was performed at 24 h, 5 days, and 10 days to evaluate cellular morphology during the differentiation of ADSCs into insulin‐producing β‐cells (Figure 4). Giemsa‐stained photomicrographs were captured using an inverted light microscope at 20× objective (Figure 4). At 24 h, Group 1 exhibited dense monolayers of evenly distributed spindle‐shaped cells with homogeneous cytoplasm and well‐defined oval nuclei. The Group 2 group displayed lower cell density and more irregular cell morphology. Among Groups 3–5 at 5 J/cm^2^, cells exposed to 525 nm showed a moderate distribution, whereas the 825 nm and combined 525 + 825 nm groups exhibited well‐spread, spindle‐shaped cells with moderate to high cell density. At 10 J/cm^2^, the 525 nm group demonstrated improved cell spreading compared with the corresponding 5 J/cm^2^ group, while the 825 nm group exhibited reduced cell density and more variable cellular morphology. The combined wavelength group maintained elongated cell morphology and moderate cell density.

At Day 5, Group 1 maintained a dense population of spindle‐shaped cells with clearly stained nuclei. In comparison, Group 2 and Groups 3–5 exhibited lower cell density and a more dispersed cellular distribution. Nuclear staining remained evident across all groups.

By Day 10, Group 1 maintained a highly confluent monolayer of elongated spindle‐shaped cells with densely stained oval nuclei. Group 2 and Groups 3–5 exhibited lower cell density and a more dispersed cellular distribution. Cellular morphology remained identifiable across all groups, with preserved nuclear staining observed throughout the experimental period.

#### 7.1.3 Dithizone (DTZ) Staining

**Figure 5:**
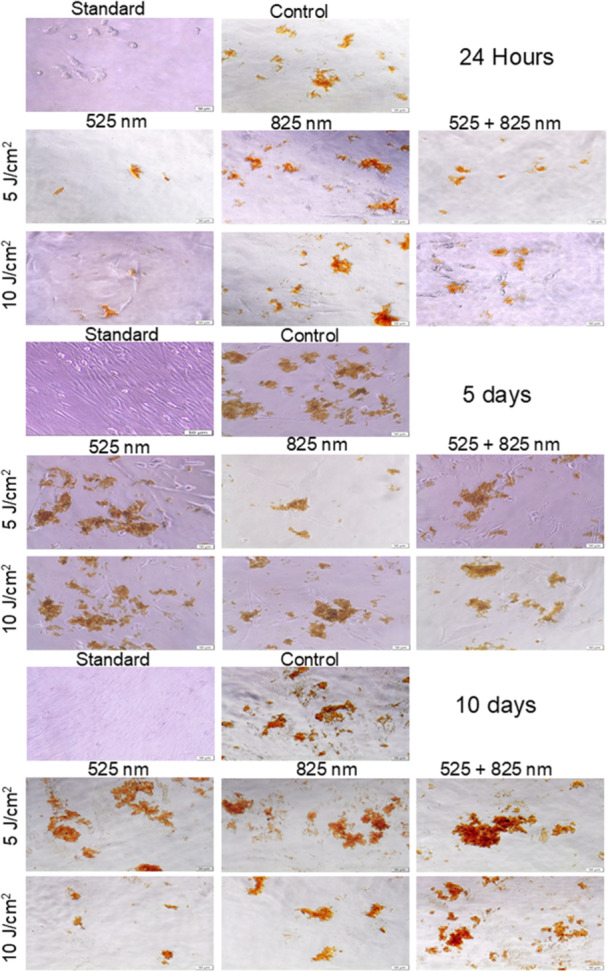
Representative photomicrographs of dithizone (DTZ)‐stained iADSCs cultured under β‐cell induction conditions and exposed to PBM at fluences of 5 and 10 J/cm^2^. Images were acquired using an inverted light microscope at 20× objective at 24 h, 5 days, and 10 days following PBM treatment. DTZ staining was used to assess the presence of zinc‐containing granules associated with β‐cell‐like differentiation during the induction process. Scale bar = 50 µM.

Dithizone (DTZ) staining was performed at 24 h, 5 days, and 10 days to evaluate the presence of zinc‐rich cell clusters during differentiation (Figure 5). DTZ‐stained photomicrographs were captured using an inverted light microscope at 20× objective (Figure 5).

At 24 h, Group 1 showed no detectable orange‐red intracellular deposits and retained a spindle‐shaped morphology. The Group 2 exhibited moderate DTZ staining distributed in scattered cellular clusters. Cells exposed to 525 nm irradiation at both 5 J/cm^2^ and 10 J/cm^2^ displayed faint DTZ staining with small dispersed clusters. In contrast, cells exposed to 825 nm irradiation exhibited greater staining intensity and a higher number of DTZ‐positive clusters. The combined 525 + 825 nm treatment produced detectable DTZ‐positive clusters at both fluences. At 5 J/cm^2^, the clusters were distributed throughout the field, whereas at 10 J/cm^2^ fewer but more densely aggregated clusters were observed.

By Day 5, DTZ‐positive clusters were evident in all treatment groups. Group 2 exhibited multiple reddish‐brown clusters. Cells exposed to 525 nm irradiation at both fluences demonstrated stronger staining intensity than the control group. Cells treated with 825 nm irradiation exhibited variable staining intensity between fluences, while the combined wavelength groups displayed intermediate staining patterns.

At day 10, Group 1 remained negative for DTZ staining, whereas the Group 2 exhibited moderate DTZ‐positive clusters. Cells exposed to 525 nm irradiation displayed prominent DTZ staining at 5 J/cm^2^, with reduced staining observed at 10 J/cm^2^. Cells exposed to 825 nm irradiation demonstrated DTZ‐positive clusters at both fluences, with a greater number of stained clusters observed at 5 J/cm^2^ than at 10 J/cm^2^. The combined 525 + 825 nm treatment produced robust DTZ staining at both fluences, with large and well‐defined DTZ‐positive clusters particularly evident at 5 J/cm^2^.

#### 7.1.4 Cell Viability: *Live/Dead Assays*

**Figure 6:**
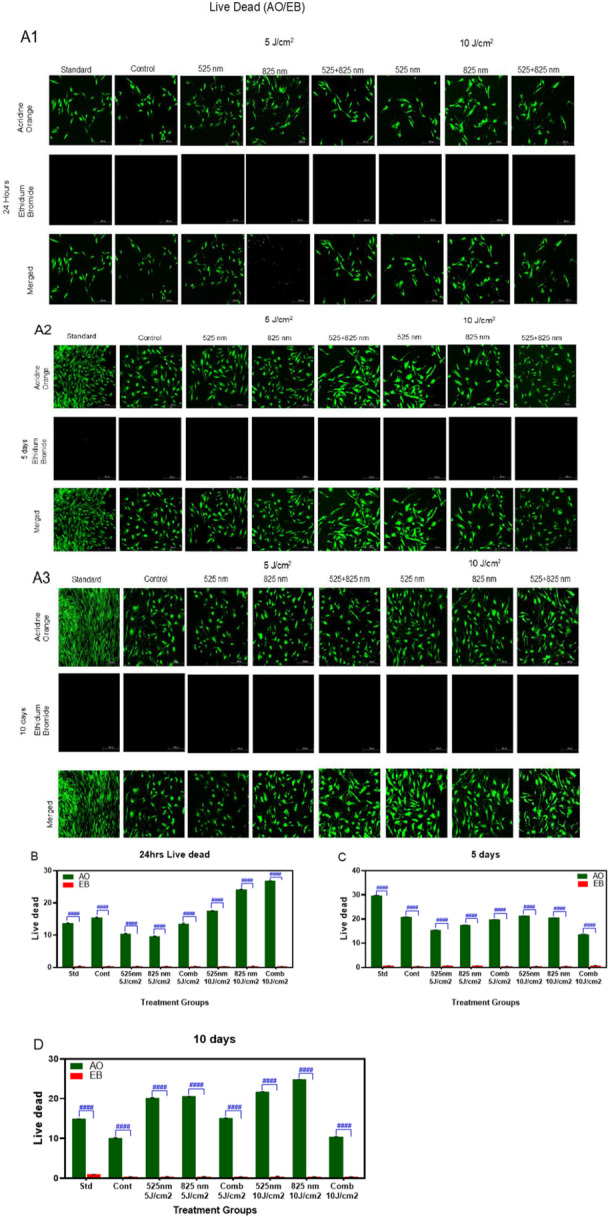
Assessment of cell viability using the Live/Dead (AO/EB) assay during β‐cell induction of iADSCs following PBM treatment. Representative photomicrographs (A1–A3) were acquired using a Leica MICA microscope at 10× objective and show Live/Dead staining of all experimental groups exposed to PBM at fluences of 5 and 10 J/cm^2^ at 24 h, 5 days, and 10 days post‐irradiation. Quantitative analysis of fluorescence intensity was performed using ImageJ, with results presented for 24 h (B), 5 days (C), and 10 days (D) post‐irradiation. Data are expressed as mean ± SEM. Statistical significance is indicated as ^#^ *p* < 0.05, ^##^ *p* < 0.01, ^###^ *p* < 0.001, and ^####^ *p* < 0.0001. AO, acridine orange; EB, ethidium bromide; Comb, combination; Cont, induction control; Std, baseline control. Scale bar = 250 µm.

Cell viability was assessed using an AO/EB Live/dead assay at 24 h, 5 days, and 10 days following PBM treatment (Figure 6A1–A3,B–D). AO/EB‐stained cells were examined using a Leica MICA microscope at 10× objective. Acridine orange stains viable cells green, whereas ethidium bromide stains membrane‐compromised cells orange to red.

At 24 h (Figure 6A1), cells in Group 1, Group 2 and Groups 3–5 predominantly exhibited green fluorescence with minimal ethidium bromide staining. Similar staining patterns were observed across all wavelengths and fluences, indicating a high proportion of viable cells.

At Day 5 (Figure 6A2), strong green fluorescence remained evident in Group 1, Group 2 and Groups 3–5. Red or orange fluorescence was minimal or absent in most fields examined. Comparable staining patterns were observed among cells exposed to 525 nm, 825 nm, and combined wavelength treatments at both 5 J/cm^2^ and 10 J/cm^2^.

By Day 10 (Figure 6A3), green fluorescence remained predominant in all experimental groups. Ethidium bromide staining remained minimal, with little evidence of extensive membrane‐compromised cells. Similar viability patterns were observed across all treatment conditions throughout the experimental period.

### 7.2 Biochemical Assays

#### 7.2.1 Proliferation: ATP Luminescence Assay

**Figure 7:**
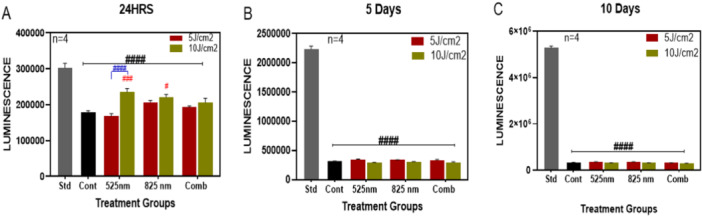
ATP luminescence assay showing cellular metabolic activity of iADSCs cultured under β‐cell induction conditions following PBM treatment at fluences of 5 and 10 J/cm^2^. ATP levels were measured at 24 h (A), 5 days (B), and 10 days (C) post‐irradiation. Data are presented as mean ± SEM. Statistical significance is indicated as ^#^ *p* < 0.05, ^###^ *p* < 0.001, and ^####^ *p* < 0.0001. Black symbols (#) indicate comparisons with the baseline control group, red symbols (#) indicate comparisons with the induction control group, and blue symbols (#) indicate comparisons between experimental groups. Comb, combination PBM group; Cont, induction control group; Std, baseline control group.

Intracellular ATP levels were measured to assess cellular metabolic activity following PBM treatment. At 24 h (Figure 7A), Group 1 exhibited significantly higher ATP levels than Group 2 and Groups 3 – 5 at both 5 J/cm^2^ and 10 J/cm^2^. Among Groups 3 – 5, cells exposed to 525 nm and 825 nm at 10 J/cm^2^ exhibited significantly higher ATP levels than the Group 2. Furthermore, cells exposed to 525 nm at 10 J/cm^2^ demonstrated significantly higher ATP levels than the corresponding 5 J/cm^2^ treatment group.

At days 5 and 10 (Figure 7B,C), ATP levels in Group 2 and Groups 3 – 5 were significantly lower than those observed in the Group 1.

#### 7.2.2 Cytotoxicity: Lactate Dehydrogenase

**Figure 8:**
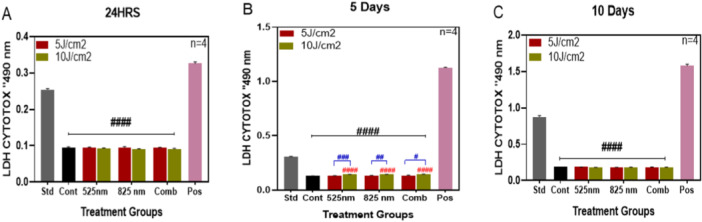
Lactate dehydrogenase (LDH) activity assay showing cytotoxicity and cell membrane integrity of iADSCs cultured under β‐cell induction conditions following PBM treatment at fluences of 5 and 10 J/cm^2^. LDH release, expressed relative to the positive control, was measured at 24 h (A), 5 days (B), and 10 days (C) post‐irradiation. Data are presented as mean ± SEM. Statistical significance is indicated as ^#^ *p* < 0.05, ^##^ *p* < 0.01, ^###^ *p* < 0.001, and ^####^ *p* < 0.0001. Black symbols (#) indicate comparisons with the baseline control group, red symbols (#) indicate comparisons with the induction control group, and blue symbols (#) indicate comparisons between experimental groups. Comb, combination PBM group; Cont, induction control group; Std, baseline control group; Pos, positive control group.

Lactate dehydrogenase (LDH) release was assessed as an indicator of cell membrane integrity and cytotoxicity at 24 h, 5 days, and 10 days post‐treatment. At 24 h (Figure 8A), Group 1 exhibited significantly higher LDH levels than Group 2 and Groups 3–5 at both 5 J/cm^2^ and 10 J/cm^2^. A similar trend was observed at Day 5 (Figure 8B), where LDH release remained significantly elevated in the Group 1 compared with the Group 2 and Groups 3–5. At day 5, cells exposed to 10 J/cm^2^ generally exhibited higher LDH levels than the corresponding 5 J/cm^2^ treatment groups. Furthermore, LDH release was significantly lower in cells irradiated with 525 nm, 825 nm, and the combined wavelengths at 5 J/cm^2^ compared with the corresponding 10 J/cm^2^ treatment groups (Figure 8B). By Day 10 (Figure 8C), LDH levels remained significantly higher in the Group 1 than in Group 2 and Groups 3–5. Notably, LDH release in all experimental groups remained substantially lower than that observed in the lysis buffer‐treated positive control, which represented maximum LDH release.

## 8 Discussion

The morphological differences observed among the treatment groups indicate that PBM influenced cellular behavior during the differentiation process. At 24 h, PBM‐treated cells exhibited increased cell spreading and more prominent cytoplasmic extensions compared with the Group 1 and Group 2, indicating an early cellular response to irradiation (Hamblin 2018; Crous et al. 2022). Similar morphological changes following PBM exposure have previously been reported in adipose‐derived stem cells, where light treatment was associated with altered cellular morphology and increased cellular activity (Crous et al. 2022).

By day 5, cells exposed to PBM, particularly at 5 J/cm^2^, exhibited increased cell density and more extensive cellular networks than the corresponding 10 J/cm^2^ groups. These findings are consistent with previous studies reporting wavelength‐ and fluence‐dependent effects of PBM on stem cell behavior (Hamblin 2018; Silva et al. 2024). However, the mechanisms underlying these morphological differences were not investigated in the present study.

By day 10, cells exposed to the combined 525 + 825 nm treatment exhibited dense cellular clustering and polygonal morphology, features that have previously been associated with differentiation‐related cellular changes (Dang et al. 2015; Fekrazad et al. 2016). Although these observations suggest that PBM may influence the differentiation process, additional molecular and functional analyses are required to confirm the underlying mechanisms.

Giemsa staining demonstrated that ADSCs maintained their characteristic spindle‐shaped morphology throughout the experimental period, although differences in cellular arrangement and density were observed among the treatment groups. Cells exposed to 825 nm irradiation and the combined wavelength treatment generally exhibited greater cellular density and more compact cellular organization than the control group, suggesting that PBM influenced cellular morphology during culture (Farivar et al. 2014).

Importantly, Giemsa staining primarily provides information on cellular morphology and nuclear structure. Therefore, while the observed morphological differences suggest that PBM influenced cellular behavior, Giemsa staining alone cannot confirm proliferation, differentiation, mitochondrial activation, cytoskeletal remodeling, or the underlying molecular mechanisms responsible for these changes. Further molecular and functional analyses are required to verify these effects

Dithizone staining was used to identify zinc‐rich cell clusters, which are commonly associated with insulin‐producing cell populations because of their elevated intracellular zinc content (Shiroi et al. 2002; Ohta et al. 2019). The absence of DTZ‐positive staining in the Group 1 throughout the experimental period suggests that basal culture conditions alone were insufficient to promote the formation of zinc‐rich cellular clusters.

Differences in DTZ staining intensity and cluster distribution were observed among the PBM‐treated groups. At 24 h, cells exposed to 825 nm irradiation and the combined 525 + 825 nm treatment exhibited more prominent DTZ‐positive clusters than the corresponding 525 nm groups, suggesting differences in zinc accumulation during the early stages of differentiation. By day 5, the 525 nm groups exhibited stronger DTZ staining than the control group, whereas the 825 nm groups displayed comparatively weaker staining. At day 10, prominent DTZ‐positive clusters were observed in the 525 nm (5 J/cm^2^), 825 nm (5 J/cm^2^), and combined wavelength groups, with particularly large and well‐defined clusters observed in the combined 525 + 825 nm treatment.

Variations were also observed between fluences. In several treatment groups, DTZ staining appeared more pronounced at 5 J/cm^2^ than at 10 J/cm^2^, suggesting that the cellular response to PBM may be influenced by energy density.

However, DTZ staining alone does not confirm β‐cell differentiation, insulin secretion, or cellular functionality. Therefore, additional molecular and functional analyses are required to confirm the differentiation status and functional characteristics of the cells.

The predominance of acridine orange‐positive cells and the limited ethidium bromide staining observed throughout the experimental period indicate that cell viability was maintained across all treatment groups. Similar findings have been reported in previous studies demonstrating that PBM can support cellular viability without inducing substantial cytotoxic effects in stem cell populations (Abrahamse and Crous 2024; Mulaudzi et al. 2026). Furthermore, comparable viability patterns were observed in both single‐wavelength and combined‐wavelength treatment groups, suggesting that the PBM parameters used in the present study did not adversely affect cell viability.

ATP is commonly used as an indicator of cellular metabolic activity and energy status (De Freitas and Hamblin 2016; Hamblin 2018). The observed differences in ATP levels among the treatment groups suggest that PBM influenced cellular metabolic activity during the experimental period. Similar PBM‐associated increases in ATP levels have been reported in adipose‐derived stem cell studies, particularly following near‐infrared irradiation (Abrahamse and Crous 2024).

Notably, ATP levels differed between Group 1, Group 2, and Groups 3–5 over time. However, the present study did not investigate the molecular mechanisms underlying these differences. Therefore, further studies are required to determine the relationship between PBM exposure, ATP production, and the differentiation process.

LDH release was used as an indicator of cell membrane integrity and cytotoxicity. Differences in LDH levels were observed among the experimental groups throughout the study period, with lower LDH levels generally observed in Groups 3–5 than in Group 1. In addition, lower LDH release was frequently observed in the 5 J/cm^2^ treatment groups compared with the corresponding 10 J/cm^2^ groups, suggesting that cellular responses varied according to the applied PBM parameters. Similar fluence‐dependent cellular responses following PBM have been reported previously in stem cell studies (De Freitas and Hamblin 2016; Dompe et al. 2020; Abrahamse and Crous 2024).

Importantly, LDH release in all experimental groups remained substantially lower than that observed in the lysis buffer‐treated positive control, indicating that the observed responses occurred within a range considerably below maximum LDH release. However, the present study did not investigate the mechanisms responsible for the observed differences in LDH levels. Therefore, further studies are required to clarify the relationship between PBM exposure, membrane integrity, and cellular responses during differentiation.

## 9 Conclusion

This study demonstrated that PBM influenced cellular responses during the in vitro differentiation of adipose‐derived stem cells (ADSCs), as evidenced by differences in cellular morphology, ATP levels, LDH release, cell viability, and DTZ staining patterns among the treatment groups. Among the investigated conditions, the combined 525 + 825 nm treatment at 5 J/cm^2^ was generally associated with favorable morphological characteristics, sustained cell viability, and prominent DTZ‐positive cluster formation throughout the experimental period. The findings further indicate that PBM parameters, including wavelength and fluence, can influence cellular responses during ADSC differentiation under in vitro conditions.

Overall, the findings of this study demonstrate that PBM influenced cellular responses during the in vitro differentiation of ADSCs, as evidenced by differences in morphology, ATP levels, LDH release, and DTZ staining patterns among the treatment groups. These findings warrant further investigation to clarify the underlying mechanisms and functional significance of these effects in stem cell‐based research and regenerative medicine.

## 10 Future Studies

Several limitations of the present study should be addressed in future investigations. First, the use of immortalized ADSCs may not fully reflect the biological characteristics of primary human ADSCs, particularly those derived from diabetic donors. Future studies should therefore evaluate the effects of PBM on primary ADSCs obtained from both healthy and diabetic individuals to improve the translational relevance of the findings.

Second, although morphological, viability, metabolic, and DTZ staining assessments were performed, additional molecular and functional analyses are required to confirm β‐cell differentiation and functionality. Future studies should incorporate gene and protein expression analyses, immunocytochemistry, Western blotting, insulin secretion assays, and ELISA‐based quantification of β‐cell‐specific markers.

Furthermore, mechanistic studies are required to investigate the molecular pathways involved in PBM‐mediated cellular responses, including signaling pathways associated with metabolism, differentiation, and cellular function. Future work should also include cytoskeletal and mitochondrial assessments using appropriate fluorescence and confocal imaging approaches.

Finally, additional PBM dosimetry studies investigating a broader range of wavelengths and fluences, together with improved optical characterization of the irradiation system and light transmission through culture media, are warranted to optimize treatment parameters and better understand the biological effects of PBM during stem cell differentiation.

## Author Contributions

**O. Daramola** and **A. Crous:** conceptualization. **O. Daramola** and **A. Crous:** writing – original draft. **O. Daramola, H. Abrahamse** and **A. Crous:** writing – review and editing. **O. Daramola:** visualization. **A. Crous** and **H. Abrahamse:** supervision. **A. Crous** and **H. Abrahamse:** funding acquisition. All authors read and approved the final manuscript.

## Ethics Statement

This study was conducted in accordance with institutional guidelines and was reviewed and approved by the University of Johannesburg Research Ethics Committee under the guidance of the NHREC. Ethical approval was granted under reference number REC‐2670‐2024. No human participants or human tissue samples were directly involved in this study. Experiments were performed using the commercially available immortalized human adipose‐derived mesenchymal stem cell line ASC52telo (hTERT) (ATCC SCRC‐4000). The cell line was obtained from the American Type Culture Collection (ATCC), where donor tissue acquisition and cell line establishment were conducted under appropriate ethical procedures and informed consent. Therefore, additional ethical approval and participant consent were not required for this study.

## Conflicts of Interest

The authors declare no conflicts of interest.

## Data Availability

The data that support the findings of this study are available from the corresponding author upon reasonable request.
